# Socioeconomic status as a predictor of post-operative mortality and outcomes in carotid artery stenting vs. carotid endarterectomy

**DOI:** 10.3389/fcvm.2024.1286100

**Published:** 2024-02-07

**Authors:** Jigesh Baxi, Joshua C. Chao, Krish Dewan, NaYoung K. Yang, Russell J. Pepe, Xiaoyan Deng, Fady K. Soliman, Lindsay Volk, Saum Rahimi, Mark J. Russo, Leonard Y. Lee

**Affiliations:** ^1^ Division of Cardiothoracic Surgery, Department of Surgery, Robert Wood Johnson University Hospital, New Brunswick, NJ, United States; ^2^ Rutgers Robert Wood Johnson Medical School, Rutgers University, New Brunswick, NJ, United States; ^3^ School of Arts and Sciences, Rutgers University, New Brunswick, NJ, United States; ^4^ Division of Vascular Surgery, Department of Surgery, Robert Wood Johnson University Hospital, New Brunswick, NJ, United States

**Keywords:** carotid artery stenting (CAS), outcomes, socioeconomic status (SES), carotid endarterectomy (CEA), carotid artery stenosis

## Abstract

**Background:**

The association between low socioeconomic status (SES) and worse surgical outcomes has become an emerging area of interest. Literature has demonstrated that carotid artery stenting (CAS) poses greater risk of postoperative complications, particularly stroke, than carotid endarterectomy (CEA). This study aims to compare the impact of low SES on patients undergoing CAS vs. CEA.

**Methods:**

The National Inpatient Sample (NIS) was queried for patients undergoing CAS and CEA from 2010 to 2015. Patients were stratified by highest and lowest median income quartiles by zip code and compared through demographics, hospital characteristics, and comorbidities defined by the Charlson Comorbidity Index (CCI). Primary outcome was in-hospital mortality. Secondary outcomes included acute kidney injury (AKI), post-operative stroke, sepsis, and bleeding requiring reoperation.Multivariable logistic regression was used to determine the effect of SES on outcomes.

**Results:**

Five thousand four hundred twenty-five patients underwent CAS (Low SES: 3,516 (64.8%); High SES: 1,909 (35.2%) and 38,399 patients underwent CEA (Low SES: 22,852 (59.5%); High SES: 15,547 (40.5%). Low SES was a significant independent predictor of mortality [OR = 2.07 (1.25–3.53); *p* = 0.005] for CEA patients, but not for CAS patients [OR = 1.21 (CI 0.51–2.30); *p* = 0.68]. Stroke was strongly associated with low SES, CEA patients (Low SES = 1.5% vs. High SES = 1.2%; *p* = 0.03), while bleeding was with high SES, CAS patients (Low SES = 5.3% vs. High SES = 7.1%; *p* = 0.01). CCI was a strong predictor of mortality for both procedures [CAS: OR1.45 (1.17–1.80); *p* < 0.001. CEA: OR1.60 (1.45–1.77); *p* < 0.001]. Advanced age was a predictor of mortality post-CEA [OR = 1.03 (1.01–1.06); *p* = 0.01]. While not statistically significant, advanced age and increased mortality trended towards a positive association in CAS [OR = 1.05 (1.00–1.10); *p* = 0.05].

**Conclusions:**

Low SES is a significant independent predictor of post-operative mortality in patients who underwent CEA, but not CAS. CEA is also associated with higher incidence of stroke in low SES patients. Findings demonstrate the impact of SES on outcomes for patients undergoing carotid revascularization procedures. Prospective studies are warranted to further evaluate this disparity.

## Introduction

The impact of socioeconomic status (SES) on clinical outcomes has become an emerging area of interest. Numerous studies published within the last two decades have reported the relationship between SES and clinical outcomes across surgical specialties. Low SES has been linked to higher postoperative mortality and increased hospital length of stay (1–5). This relationship has been demonstrated across international cohorts and been linked to higher postoperative complications with patients undergoing oncologic surgery.

According to the Pew Research Center, economic inequality–whether defined by the gap in income or in wealth between higher and lower income households–has continued to widen in the US since 2009. In this context, efforts to understand the relationship between income inequality and health outcomes has become imperative. As of 2020, the Centers for Disease Control and Prevention (CDC) estimates that 696,962 deaths in the US could be attributed to cardiovascular disease (CVD), making it the leading cause of death in this country (6, 7). Efforts must be made to understand how social determinants of health impact patients receiving revascularization procedures. Prior studies have highlighted how race and low SES influence rates of major amputation in peripheral vascular disease and correlate with delay of care in coronary artery disease (CAD) and myocardial infarction (MI) (1, 8). More recent studies have shown the deleterious impact of low SES for patients undergoing carotid artery stenting (CAS) and carotid endarterectomy (CEA) (9, 10). These studies were confounded by several demographic variables: race, access to healthcare, underlying patient comorbidities, and several patient-specific underlying comorbidities. There remains a need to better understand the independent relation of SES and the outcomes of carotid revascularization procedures. The purpose of the present study is to determine the independent association of SES and post-operative outcomes for patients undergoing elective carotid revascularization using a real-world large national database.

## Methods

### Data source

Data for this project were obtained from the National Inpatient Sample (NIS), a database overseen by the *Healthcare Cost and Utilization Project (HCUP)* from the Agency for Healthcare Research and Quality. Users can purchase the data after completing the HCUP Data Use Agreement and associated training. Data are protected for legal and ethical purposes. Currently, the NIS represents the most comprehensive all-payor database of hospital discharges in the US encompassing approximately twenty percent of non-federal US hospitals, including specialty and academic institutions (11), and expanding beyond cancer registries per other well-known databases. The NIS years 2010 through 2015 were queried to identify patients undergoing CEA and CAS. Each procedure was analyzed and modelled independently.

### Patient population

Patients were excluded from the analysis if they were less than 18 years of age or were missing data on mortality or median household income by zip code. Patients who underwent the procedure >3 days after admission were excluded to minimize the possibility of including non-elective cases. Patients were then further stratified by SES as defined by the median income of patients' reported zip codes (the lowest and highest quartile of median zip code income defined as low and high SES, respectively).

### Statistical analysis

Post-procedural outcomes were compared between high and low SES for each procedure. The primary outcome was defined as in-hospitality mortality. Secondary outcomes included acute kidney injury (AKI), post-operative stroke, sepsis, and bleeding requiring reoperation.

For each procedure, univariable analysis was conducted to compare covariates such as patient demographics, comorbidities (Charlson comorbidity index factors), and hospital characteristics (hospital teaching status, region, and bed size). Prior to analysis, variables were checked for normal data distribution using graphical representation of data through histograms. Given parametric distribution, *t*-tests were chosen as mode of univariable data analysis. Categorical variables were summarized as counts and proportions; these were compared using Pearson chi-square test. Continuous variables were summarized as means and compared using the student *t*-test. One-way ANOVA test was used to compare numeric and categorical variables for each procedure.

Multivariable logistic regression modelling was utilized to measure the association between the two SES categories and the primary and secondary outcomes, accounting for patient demographics, predisposing conditions, and hospital characteristics. Test was chosen based on the binary nature of the primary outcome. Variables for the regression were chosen based on combination of statistical significance (*p* < 0.05) in the initial univariable analysis and *a priori* clinical relevance to outcome variables of interest. Results are presented as odds ratios with a 95% confidence interval (CI). Covariates in the multivariable model were selected from the list of independent variables considered for each procedure. All analyses were performed using SAS 9.4 M7 software (SAS Institute, Cary, North Carolina) (12).

## Results

A total of 43,824 patients who underwent CEA and CAS between 2010 and 2015 were available as reported by the NIS database. After applying our exclusion criteria, 5,425 patients underwent CAS, of whom 3,516 (64.8%) patients were of low SES and 1,909 (35.2%) patients were of high SES. Of the 38,399 patients who underwent CEA, 22,852 (59.5%) patients were of low SES and 15,547 (40.5%) patients were of high SES.

Between both procedures, low SES patients tended to be younger. In the CAS group, the average age was 69.9 ± 9.9 years in the low SES subgroup vs. 71.4 ± 10.0 years in the high SES subgroup. In the CEA group, the average age was 70.3 ± 9.3 years in the low SES subgroup vs. 72.0 ± 9.1 years in the high SES subgroup (*p* < 0.001). A greater proportion of low SES patients was female in both CAS [1,457 (41.4%) female vs. 2,059 (38.3%) male; *p* = 0.03] and in CEA [10,000 (43.8%) female vs. 12,852 (38.6%) male; *p* < 0.001] groups. In both procedures, there were significant differences with race and payer status. The majority of patients undergoing both procedures in both the low and high SES groups were White; however, a higher portion of non-White patients were seen in the low SES group with both procedures [CAS: low SES 2,886 (82.1%) vs. high SES 1,674 (87.7%), *p* < 0.001; CEA: low SES 19,032 (83.3%) vs. high SES 13,885 (89.3%), *p* < 0.001]. Notably, Medicare recipients comprised a smaller portion of patients undergoing CAS in the lower SES subgroup. When compared with patients of high SES undergoing CAS and with patients of both low and high SES undergoing CEA, patients with low SES undergoing CAS were much less likely to be Medicare recipients [CAS low SES 1,656 (47.1%) vs. high SES 1,373 (71.9%), *p* < 0.001; CEA low SES 17,005 (74.4%) vs. high SES 11,413 (73.4%), *p* < 0.001]. With respect to comorbidities, lower SES was associated with significantly more comorbidities across both procedures. Compared to their respective high SES counterparts, lower SES patients undergoing CAS had a higher incidence of congestive heart failure [Low SES 451 (12.8%) vs. High SES 185 (9.7%); *p* < 0.001], cerebral vascular accident [Low SES 3,493 (99.3%) vs. High SES 1,880 (98.5%); *p* = 0.003], pulmonary disease [Low SES 934 (26.6%) vs. High SES 334 (17.5%); *p* < 0.001], and diabetes [Low SES 1,151 (32.7%) vs. 533 (27.9%); *p* < 0.001]. Meanwhile, low SES patients undergoing CEA had a higher incidence of acute myocardial infarction [Low SES 3,041 (13.3%) vs. High SES 1,854 (11.9%); *p* < 0.001], congestive heart failure [Low SES 1,813 (7.9%) vs. 1,067 (6.9%); *p* < 0.001], peripheral vascular disease [Low SES 5,501 (24.1%) vs. High SES 3,546 (22.8%); *p* = 0.004], pulmonary disease [Low SES 5,683 (24.9%) vs. High SES 2,889 (18.6%); *p* < 0.001], connective tissue disorder [Low SES 118 (0.5%) vs. High SES 1,067 (6.9%); *p* < 0.001], liver disease [Low SES 6,304 (27.6%) vs. High SES 3,824 (24.6%); *p* < 0.001], and diabetes [Low SES 7,468 (32.7%) vs. High SES 4,250 (27.3%); *p* < 0.001]. Table 1 summarizes the baseline characteristics of each group.

**Table 1:** Baseline and clinical characteristics of patient groups.

Characteristic	Carotid artery stenting (CAS)			Carotid endarterectomy (CEA)		
	Low SES (*n* = 3,516)	High SES (*n* = 1,909)	*p*-value (*α* = 0.05)	Low SES (*n* = 22,852)	High SES (*n* = 15,547)	*p*-value (*α* = 0.05)
Baseline demographics						
Age (years) (mean ± SD)	69.9 ± 9.9	71.4 ± 10.0	<0.001	70.3 ± 9.3	72.0 ± 9.1	<0.001
Female *n* (%)	1,457 (41.4%)	731 (38.3%)	0.0259	10,000 (43.8%)	5,997 (38.6%)	<0.001
Race *n* (%)						
Caucasian	2,886 (82.1%)	1,674 (87.7%)	<0.001	19,032 (83.3%)	13,885 (89.3%)	<0.001
African-American	335 (9.5%)	76 (4.0%)		2,129 (9.3%)	540 (3.5%)	
Hispanic	187 (5.3%)	55 (2.9%)		1,033 (4.5%)	401 (2.6%)	
Asian or Pacific Islander	20 (0.6%)	55 (2.9%)		91 (0.4%)	317 (2.0%)	
Native American	24 (0.7%)	3 (0.2%)		178 (0.8%)	33 (0.2%)	
Other	64 (1.8%)	46 (2.4%)		389 (1.7%)	371 (2.4%)	
Patient insurance *n* (%)						
Medicare	2,656 (75.5%)	1,373 (71.9%)	<0.001	17,005 (74.4%)	11,413 (73.4%)	<0.001
Medicaid	195 (5.5%)	58 (3.0%)		1,117 (4.9%)	297 (1.9%)	
Private including HMO	511 (14.5%)	435 (22.8%)		4,012 (17.6%)	3,576 (23.0%)	
Self-pay	64 (1.8%)	9 (0.5%)		336 (1.5%)	100 (0.6%)	
No charge	6 (0.2%)	0 (0.0%)		42 (0.2%)	8 (0.1%)	
Other	84 (2.4%)	34 (1.8%)		340 (1.4%)	153 (1.0%)	
Clinical characteristics *n* (%)						
Acute myocardial infarction	455 (12.9%)	250 (13.1%)	0.9046	3,041 (13.3%)	1,854 (11.9%)	<0.001
Atrial fibrillation	355 (10.1%)	236 (12.4%)	0.012	1,932 (8.5%)	1,721 (11.1%)	<0.001
Cancer	53 (1.5%)	43 (2.3%)	0.0601	237 (1.0%)	213 (1.4%)	0.0034
Cerebral vascular accident	3,493 (99.3%)	1,880 (98.5%)	0.0029	22,810 (99.8%)	15,530 (99.9%)	0.09
Congestive heart failure	451 (12.8%)	185 (9.7%)	<0.001	1,813 (7.9%)	1,067 (6.9%)	<0.001
Connective tissue disorder	9 (0.3%)	9 (0.5%)	0.2843	118 (0.5%)	120 (0.8%)	0.0022
COPD	1,358 (38.6%)	569 (29.8%)	<0.001	9,229 (40.4%)	5,261 (33.8%)	<0.001
Coronary artery disease	318 (9.0%)	171 (9.0%)	0.9546	1,343 (5.9%)	796 (5.1%)	0.0016
Dementia	5 (0.1%)	4 (0.2%)	0.7285	29 (0.1%)	23 (0.1%)	0.6827
Diabetes	1,151 (32.7%)	533 (27.9%)	<0.001	7,468 (32.7%)	4,250 (27.3%)	<0.001
Diabetes complications	81 (2.3%)	47 (2.5%)	0.7848	674 (2.9%)	516 (3.3%)	0.0433
Dyslipidemia	1,978 (56.3%)	1,078 (56.5%)	0.903	10,698 (46.8%)	7,792 (50.1%)	<0.001
HIV	0 (0.0%)	0 (0.0%)	1	0 (0.0%)	0 (0.0%)	1
Hypertension	2,449 (69.7%)	1,304 (68.3%)	0.3203	16,676 (73.0%)	11,342 (73.0%)	0.9732
Liver disease	794 (22.6%)	402 (21.1%)	0.208	6,304 (27.6%)	3,824 (24.6%)	<0.001
Metastatic cancer	5 (0.1%)	4 (0.2%)	0.7285	26 (0.1%)	26 (0.2%)	0.2088
Paraplegia	2 (0.1%)	0 (0.0%)	0.5438	7 (0.0%)	4 (0.0%)	1
Peptic ulcer	17 (0.5%)	7 (0.4%)	0.6855	127 (0.6%)	79 (0.5%)	0.5783
Peripheral vascular disease	1,083 (30.8%)	572 (30.0%)	0.542	5,501 (24.1%)	3,546 (22.8%)	0.0043
Prior CABG	665 (18.9%)	353 (18.5%)	0.7309	3,996 (17.5%)	2,636 (17.0%)	0.1807
Prior PCI	531 (15.1%)	277 (14.5%)	0.5857	2,812 (12.3%)	1,905 (12.3%)	0.8912
Pulmonary disease	934 (26.6%)	334 (17.5%)	<0.001	5,683 (24.9%)	2,889 (18.6%)	<0.001
Renal disease	384 (10.9%)	239 (12.5%)	0.0857	2,229 (9.8%)	1,601 (10.3%)	0.084
Severe liver disease	3 (0.1%)	0 (0.0%)	0.5565	5 (0.0%)	3 (0.0%)	1
Hospital status *n* (%)						
Rural	259 (7.4%)	4 (0.2%)	<0.001	3,852 (16.9%)	67 (0.4%)	<0.001
Urban non-teaching	1,034 (29.4%)	663 (34.7%)		7,991 (35.0%)	6,258 (40.3%)	
Urban teaching	2,223 (63.2%)	1,242 (65.1%)		11,009 (48.2%)	9,222 (59.3%)	
Hospital region *n* (%)						
Northeast	286 (8.1%)	465 (24.4%)	<0.001	1,658 (7.3%)	4,573 (29.4%)	<0.001
Midwest	652 (18.5%)	392 (20.5%)		4,863 (21.3%)	3,094 (19.9%)	
South	2,217 (63.1%)	612 (32.1%)		14,063 (61.5%)	4,349 (28.0%)	
West	361 (10.3%)	440 (23.0%)		2,268 (9.9%)	3,531 (22.7%)	
Hospital bed size *n* (%)						
Small	277 (7.9%)	246 (12.9%)	<0.001	1,994 (8.7%)	2,019 (13.0%)	<0.001
Medium	1,043 (29.7%)	370 (19.4%)		5,221 (22.8%)	3,939 (25.3%)	
Large	2,196 (62.5%)	1,293 (67.7%)		15,637 (68.4%)	9,589 (61.7%)	

In-hospital mortality was low across both procedures, but worse for low SES patients vs. high SES patients undergoing CEA. This was not seen when comparing low SES to high SES patients undergoing CAS. In this study, mortality was observed at 0.4% in the low SES/CAS subgroup vs. 0.4% in the high SES/CAS subgroup (*p* = 0.91) and 0.3% in the low SES/CEA subgroup vs. 0.1% in the high SES/CEA subgroup (*p* = 0.005).

**Figure 1:**
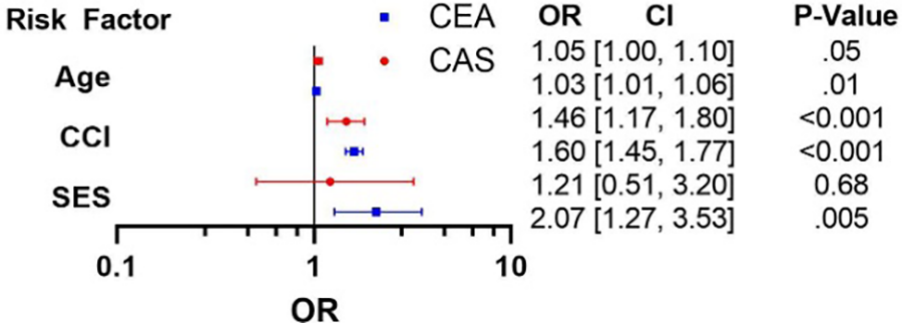
Independent predictors of mortality.

Using multivariable logistic regression, we identified independent predictors of in-hospital mortality (Figure 1) after controlling for patient demographics, comorbidities, and hospital characteristics. Variables were chosen for the logistic regression based on statistical significance (*p* < 0.05) seen in univariable analysis (Table 1) and *a priori* selection. Low SES was a significant independent predictor of mortality for patients who underwent a CEA [OR = 2.07 (1.25–3.53), *p* = 0.005] but not for those undergoing CAS. CCI and age were strong predictors of mortality for both procedures [CAS: OR_age_ 1.05 (1.00–1.10), *p* = 0.05; OR_CCI_ 1.45 (1.17–1.80) *p* < 0.001. CEA: OR_age_ 1.03 (1.01–1.06), *p* = 0.01; OR_CCI_ 1.60 (1.45–1.77), *p* < 0.001].

**Figure 2:**
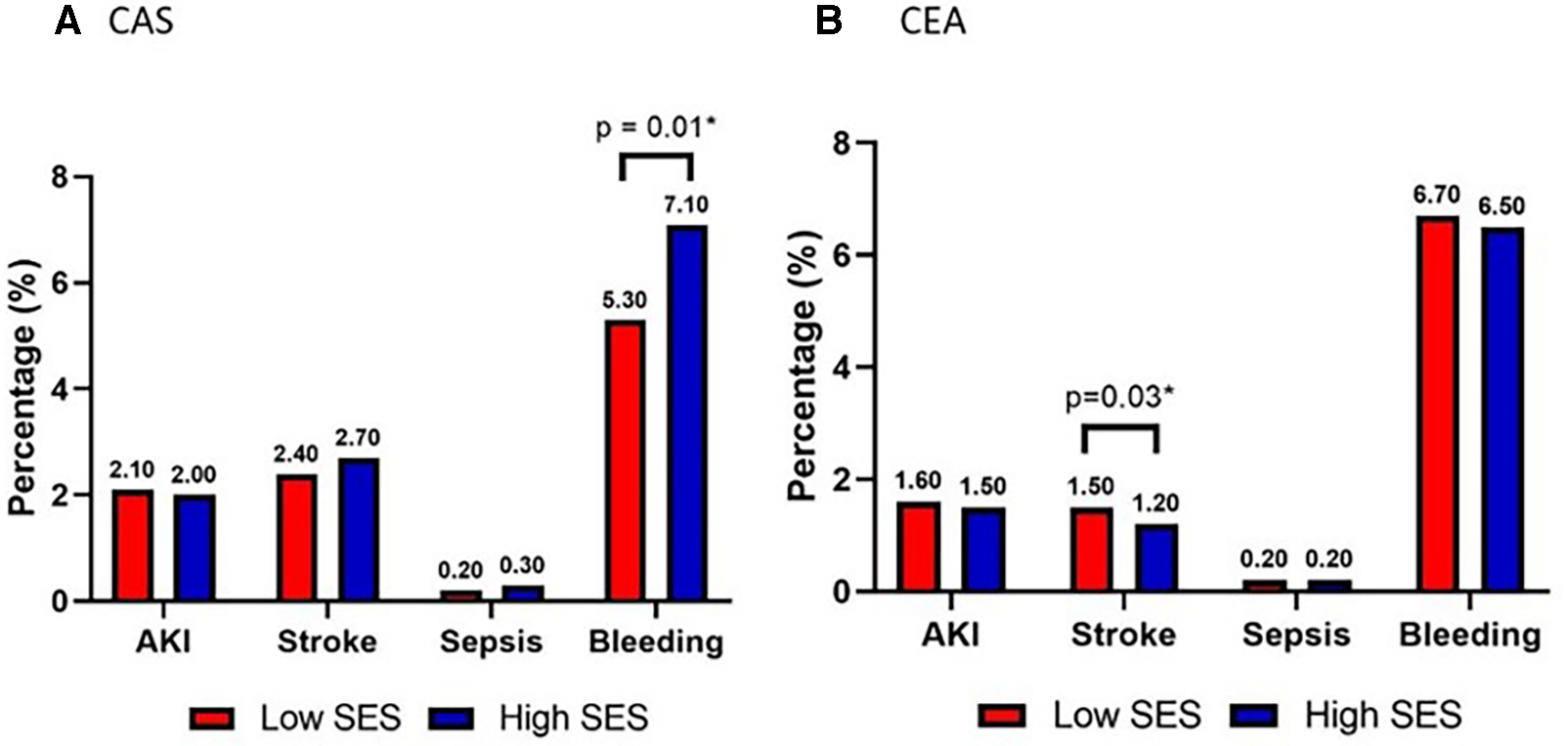
Secondary outcomes acute kidney injury (AKI), stroke, sepsis, bleeding requiring reoperation in patients stratified by low (red) vs. high (blue) socioeconomic status who underwent (**A**) carotid artery stenting (CAS) or (**B**) carotid endarterectomy (CEA).

Post procedure complications (secondary outcomes) are demonstrated in Table 2; the most frequent outcomes are depicted graphically in Figure 2. Among these outcomes, a higher incidence of stroke was strongly associated with low SES patients who underwent CEA (Low SES = 1.5% vs. High SES = 1.2%; *p* = .03). Bleeding was associated with high SES patients undergoing CAS (Low SES = 5.3% vs. High SES = 7.1%; *p* = .01).

**Table 2:** Postoperative outcomes and characteristics.

Outcome	Carotid artery stenting (CAS)			Carotid endarterectomy (CEA)		
	Low SES (*n* = 3,516)	High SES (*n* = 1,909)	*p*-value (*α* = 0.05)	Low SES (*n* = 22,852)	High SES (*n* = 15,547)	*p*-value (*α* = 0.05)
Stroke	83 (2.4%)	51 (2.7%)	0.5399	**332** (**1.5%)**	**186** (**1.2%)**	**0**.**0363**
Acute kidney injury	75 (2.1%)	39 (2.0%)	0.9029	361 (1.6%)	227 (1.5%)	0.3709
Left bundle branch block	24 (0.7%)	7 (0.4%)	0.1986	**110** (**0.5%)**	**104** (**0.7%)**	**0**.**0186**
Complete heart block	22 (0.6%)	13 (0.7%)	0.9479	134 (0.6%)	116 (0.7%)	0.0649
Bleeding	**188** (**5.3%)**	**135** (**7.1%)**	**0**.**0123**	1,520 (6.7%)	1,011 (6.5%)	0.5787
Shock	3 (0.1%)	6 (0.3%)	0.0749	16 (0.1%)	15 (0.1%)	0.4757
Sepsis	7 (0.2%)	6 (0.3%)	0.3992	48 (0.2%)	25 (0.2%)	0.3330
Delirium	7 (0.2%)	5 (0.3%)	0.7635	38 (0.2%)	24 (0.2%)	0.8760
Acute pulmonary edema/failure	39 (1.1%)	20 (1.0%)	0.9429	**268** (**1.2%)**	**130** (**0.8%)**	**0**.**0017**
Surgical site infection	4 (0.1%)	2 (0.1%)	1.0000	16 (0.1%)	13 (0.1%)	0.7741
Pneumonia	33 (0.9%)	23 (1.2%)	0.4319	372 (1.6%)	244 (1.6%)	0.6848

The bolded values emphasise variables with significant *p*-value <0.05.

## Discussion

This study demonstrates the association between SES and postoperative outcomes for patients undergoing carotid procedures utilizing a nationally representative database. Our results indicated that SES is associated with postoperative mortality for patients who underwent CEA independent of demographic factors including race, age, gender, or CCI, or in-hospital characteristics, while low SES was correlated with higher rates of postoperative stroke for patients undergoing CEA but not CAS. Our study also demonstrates that baseline comorbidity burden is a strong prognostic factor for postoperative mortality, controlling for other baseline demographic factors.

The correlation between SES and adverse outcomes for patients undergoing CEA that we have demonstrated is supported by prior studies. Vogel et al., similarly demonstrated an increase in mortality (OR = 1.64, CI 1.25–2.14) with patients who had carotid procedures (CEA and CAS) in the lowest income quartile using the NIS (10). This study, however, examined carotid procedures as a single composite cohort. Our group assessed CEA and CAS as two distinct cohorts. Prior studies have demonstrated a correlation between higher rates of comorbidities with either race or low socioeconomic status, which often confounds measured outcomes (13). While the results of our univariable analysis would likely have been confounded by these same demographic associations, by controlling for baseline comorbidities and incorporating the CCI into a multivariable logistic regression model, we were able to demonstrate the adverse impact of SES independent of CCI.

Multiple factors may contribute to defining the adverse relationship between low SES and clinical outcomes following CEA. Education, which is only one component of the complex state that makes up low SES, has been correlated with higher rates of risk factors for cardiovascular disease (14). Despite overall improvements in awareness and education in cardiovascular risk factors in recent decades in the US, Americans in the lowest SES subpopulations have shown a higher prevalence of smoking and diabetes relative to the higher SES population (15). In a recent large-scale multinational prospective study conducted in high income countries, both low income and limited education were associated with higher prevalence of well-established cardiovascular risk factors, including hypertension and diabetes, which resulted in higher incidence of cardiovascular mortality (16). Other studies have demonstrated clinical relevance of this sociomedical phenomenon by showing that patients of lower SES not only have higher rates of atherosclerotic risk factors, but also increased intima-media thickness (IMT) measurements within arteries and delayed recovery in cardiovascular function after mental stress (17, 18). Still, our current study found an independent association between SES and mortality even when adjusting for baseline demographics and comorbidities; therefore, comorbidities alone do not explain disparities in outcomes seen between SES strata.

By examining this subset of patients with low SES, our study may help inform patient selection for either CEA or CAS when taken in context of existing literature. Notably, in our study, we observed an increase in the rate of postoperative stroke amongst low SES patients who underwent CEA but not for CAS. The difference in stroke rates after either procedure is still a point of active discussion. The 2010 Carotid Revascularization Endarterectomy vs. Stenting Trial (CREST) concluded that CAS and CEA had similar short and long-term outcomes, despite higher risks of postoperative stroke with CAS (19). However, several subsequent studies and subgroup analyses have called into question the conclusion of equivalence posed by the CREST trial due to (1) the trial's inclusion of a heterogeneous population of symptomatic and asymptomatic patients, and (2) the false equivalence of minor MI (more prevalent after CEA) with stroke (more prevalence after CAS) (20–22). Subgroup analyses of symptomatic patients were the only group of patients where stroke rate did not differ between CEA and CAS, consistent with the results of a more recent randomized multinational clinical trial, the Second Asymptomatic Carotid Surgery Trial (ACST-2) (20).

Hence, the 2021 Society for Vascular Surgery guidelines generally favour CEA over CAS in most cases, with careful consideration of CAS for patients with special clinical circumstances or high operative risk (23). For this reason, CAS is generally reserved for patients with operative fields in the neck surgically unfit for CEA, e.g., reoperative or irradiated fields. Our results indicating a correlation between periprocedural stroke and low SES after CEA but not for CAS raises an important question that should become a focus of future studies—do well-selected patients of low SES benefit from CAS over CEA? Limited retrospective data suggest that patients with low SES are more likely to undergo CAS, which some have suggested may explain outcome disparities (24, 25). Given the limitations of administrative data and the complex associations between socioeconomic status, race, and preoperative medical risk, it is not possible from this study to determine whether the use of CAS was limited to well-selected high-risk patients with low SES, or whether there is a true benefit to utilization of CAS in this subgroup over CEA.

Estimating perioperative risk is a key component during surgical planning. Various tools exist that estimate peri-operative risks from baseline disease burden, however few of these tools incorporate SES. Our study demonstrates the importance of considering SES when evaluating pre-operative risk assessment. These findings highlight the necessity to further evaluate the difference in outcomes associated with SES and patients undergoing CES and CAS. Further studies should be done to delineate the relationship between SES and these outcomes and to further characterize components of SES other than income that may correlate with disparities in care. Studies that involve analysis of the relationship between race and ethnicity and the zip code incomes that were used as proxy for SES in this study would help highlight the complex nature of social determinants of health on patient outcomes. Educational status and access to transportation in relation to patient's distance from hospital can also elucidate further components of SES that future studies should address when discussing SES as a whole independent variable on patient outcomes.

### Limitations

There are several limitations to our study. The NIS database introduces heterogeneity within the data, subjecting our study to coding bias. Patient SES is defined as median income of patient zip code, which may or may not reflect true SES status as this leaves out the relationship between SES and patient demographics. Our data may demonstrate “access to healthcare” rather than SES status. Mortality coding in NIS is defined as in-hospital death; however, post-discharge mortality has variable coding which would have added further heterogeneity to the analysis. There are inherent limitations to using ICD codes due to coding bias and inconsistent coding practices from institution to institution. Despite these limitations, this study offers valuable insight into the effect of SES on outcomes by harnessing data from a real-world large sample of patients undergoing vascular surgery.

## Conclusion

Low SES is a significant independent predictor of post-operative mortality in patients who undergo CEA, but not CAS, while CEA was associated with a higher incidence of stroke in these patients with low SES status. These findings demonstrate the impact of SES on outcomes for patients undergoing carotid revascularization procedures and emphasize the need for future studies to further evaluate this disparity.

## Data Availability

The data analyzed in this study is subject to the following licenses/restrictions: Patient dataset. Requests to access these datasets should be directed to RP, rpepe@rwjms.rutgers.edu.
